# Simultaneous Recording of Remote Domain Dynamics in Membrane Proteins Using the Double-Labeled DXB/DXT Technique

**DOI:** 10.3390/membranes14040075

**Published:** 2024-03-27

**Authors:** Kazuhiro Mio, Tatsunari Ohkubo, Daisuke Sasaki, Mayui Sugiura, Kayoko Kawaguchi, Kazutaka Araki, Keizaburo Taninaka, Masaki Sakaguchi, Shunsuke Nozawa, Tatsuya Arai, Yuji C. Sasaki

**Affiliations:** 1 AIST-UTokyo Advanced Operando-Measurement Technology Open Innovation Laboratory (OPERANDO-OIL), National Institute of Advanced Industrial Science and Technology (AIST), 6-2-3 Kashiwanoha, Chiba 277-0882, Japan; w225405f@yokohama-cu.ac.jp (T.O.); m.sugiura@aist.go.jp (M.S.); kawaguchi.k@aist.go.jp (K.K.); k-araki@aist.go.jp (K.A.); 3152469031@edu.k.u-tokyo.ac.jp (K.T.); 8507367711@edu.k.u-tokyo.ac.jp (M.S.); 2 Graduate School of Medical Life Science, Yokohama City University, 1-7-29 Suehiro-cho, Tsurumi-ku, Yokohama 230-0045, Japan; 3 Graduate School of Frontier Sciences, The University of Tokyo, 5-1-5 Kashiwanoha, Chiba 277-8561, Japan; 4097663473@edu.k.u-tokyo.ac.jp (D.S.); t.arai@edu.k.u-tokyo.ac.jp (T.A.); 4 Photon Factory, Institute of Materials Structure Science, High Energy Accelerator Research Organization, 1-1 Oho, Tsukuba 305-0801, Japan; noz@post.kek.jp

**Keywords:** diffracted X-ray tracking (DXT), diffracted X-ray blinking (DXB), dual-labeling technique, intramolecular dynamics, membrane proteins

## Abstract

Protein dynamics play important roles in biological functions, which accompany allosteric structure changes. Diffracted X-ray blinking (DXB) uses monochromatic X-rays and nanocrystal probes. The intramolecular motion of target proteins is analyzed from the intensity changes in detector signals at the diffraction rings. In contrast, diffracted X-ray tracking (DXT) elucidates molecular dynamics by analyzing the trajectories of Laue spots. In this study, we have developed a dual-labeling technique for DXB and DXT, allowing the simultaneous observation of motions at different domains in proteins. We identified zinc oxide (ZnO) crystals as promising candidates for the second labeling probes due to their excellent diffraction patterns, high chemical stability, and favorable binding properties with proteins. The diffraction spots from the ZnO crystals are sufficiently separated from those of gold, enabling independent motion analysis at different domains. Dual-labeling DXB was employed for the motion analysis of the 5-HT_2A_ receptor in living cells. Simultaneous motion recording of the N-terminus and the second extracellular loop demonstrated ligand-induced motion suppression at both domains. The dual-labeling DXT technique demonstrated a capsaicin-induced peak shift in the two-dimensional motion maps at the N-terminus of the TRPV1 protein, but the peak shift was not obvious in the C-terminus. The capsaicin-induced motion modulation was recovered by the addition of the competitive inhibitor AMG9810.

## 1. Introduction

The breakthrough in cryo-electron microscopy [1,2,3] and advancements in crystallization techniques [4,5,6] have made it easier to obtain atomic structures of membrane proteins that were previously challenging. However, to understand their functions, a detailed mechanism of intramolecular domain movement, including the analysis of transition-state structures and time-dependent frequencies with sufficient time-scale information, is also indispensable.

Accurate measurement of the motion of membrane proteins under sufficient spatiotemporal resolution poses a challenge. The fundamental issue has been the absence of a direct measuring method for the rapid and precise movements of such small molecules. To answer this question, we have been researching diffracted X-ray tracking (DXT) [7], a real-time measurement technique with the highest spatiotemporal resolution among currently available single-molecule measurement techniques. In DXT, gold nanocrystals are bound to specific sites on the protein, and the movement of diffraction spots under X-ray irradiation is analyzed to elucidate the motion of the protein. Its efficacy has been demonstrated in various membrane proteins, such as the KcsA potassium channel, the acetylcholine receptor, and the TRPV1 channel [8].

We have also discovered a diffracted X-ray blinking (DXB) method that quantitatively measures molecular fluctuations even with monochromatic X-rays [9]. The DXB does not track trajectories, but calculates the autocorrelation coefficient of brightness at pixels, revealing protein dynamics. Because DXB uses monochromatic X-rays instead of white or pink beams, it can be performed even using laboratory X-ray generators. The use of a low-flux energy X-ray reduces sample damage, allowing us to measure the dynamics of membrane proteins even in living cells [10,11].

To achieve the dual-labeling technique, a second labeling probe with different diffraction properties from those of gold is necessary. After careful consideration, we found that zinc oxide (ZnO) crystals were suitable for the second labeling probe because of their high-quality diffraction spots, high chemical stability, and easy binding to proteins. In this study, we introduced gold and ZnO nanocrystals into different domains of a single protein and simultaneously analyzed their dynamics. By employing the dual-labeling technique, we conducted simultaneous living-cell DXB analysis of the N-terminus and the second extracellular loop of the metabotropic serotonin receptor subtype 2A (5-HT_2A_R). The 5-HT_2A_R belongs to a G protein-coupled receptor (GPCR) family and is widely distributed in the central nervous system, playing crucial roles in neuronal excitation, learning, and emotion [12,13,14]. Using DXT and DXB techniques, we previously demonstrated ligand-induced structural convergence of the 5-HT_2A_R from a mixture of multiple forms to active or inactive states [10,11]. We also performed here dual-labeling DXT analysis of the N- and C-terminal cytoplasmic domains of the transient receptor potential vanilloid 1 (TRPV1) channel. TRPV1 serves as a multimodal receptor, reacting to a range of stimuli such as capsaicin, protons, and heat. Additionally, it holds significant importance in the perception of pain [15,16,17]. In our previous study, observations revealed that both an agonist and a competitive antagonist induced a rotational bias in TRPV1, albeit in opposite directions. Additionally, we noted a reversal in the rotational bias elicited by capsaicin between the wild-type and the capsaicin-insensitive Y511A mutant [18]. We also recently demonstrated real-time observation of capsaicin-induced fluctuation at the C-terminus and CCW twisting motion at the N-terminus of TRPV1 using DXT [19]. Through the utilization of these samples, we investigated the dual-labeling systems employed in this study.

## 2. Methods

### 2.1. Sample Preparation for Dual-Labeling Living-Cell DXB

The full-length human 5-HT_2_AR was cloned into pcDNA3.1, with a FLAG tag sequence (DYKDDDDK) [20] introduced at the N-terminus and a PA tag sequence (GVAMPGAEDDVV) [21] introduced at the second extracellular loop using the inverse PCR system (Toyobo biotech, Osaka, Japan). FreeStyle HEK 293-F cells (Thermo Fisher, Waltham, MA, USA) were cultured in FreeStyle 293 Expression Medium (Thermo Fisher) at 37 °C with 5% CO_2_. Transfection was performed by mixing 1 μg of plasmid DNA with polyethyleneimine MAX (Polysciences, Warrington, PA, USA) at a ratio of 1:3 (weight) into 1 mL of cells at a density of 2 × 10^6^ cells/mL. The cells were then dispersed in Dulbecco’s modified Eagle’s medium supplemented with 10% fetal bovine serum, 100 U/mL penicillin, and 100 μg/mL streptomycin, and inoculated onto 12.5 μm thick polyimide films (Kapton, Du Pont-Toray, Tokyo, Japan) at a density of 5 × 10^5^ cells/cm^2^. The polyimide films were treated with 0.001% poly L-lysine (Peptide institute, Osaka, Japan) in advance. Cells were further cultured for 48 h.

Gold nanocrystals with diameters ranging from 40 to 80 nm were synthesized through epitaxial growth on a KCl (100) substrate under vacuum conditions of 10^−4^ Pa. These nanocrystals were then dissolved in double-distilled water and combined with 100 μg of FLAG antibody (clone M2, Sigma-Aldrich, St. Louis, MO, USA) in 1 mL of borate buffer (pH 9.0) with vigorous vortexing. Separately, 50 μg of ZnO nanocrystals (purity > 99.9%, average diameter of 500 nm, EM-Japan, Tokyo, Japan) were washed with distilled water through sonication and were then conjugated with 100 μg of (His)_6_ monoclonal antibody (clone 9C11, FUJIFILM-Wako, Osaka, Japan) in 1 mL of borate buffer (pH 9.0) with vigorous vortexing. In both complexes, the mixtures were further dispersed with ultrasonic sonication for 30 min on ice and unbound antibodies were removed by centrifugation at ×1000 rpm for 10 min twice. Buffer was substituted with PBS (pH 7.4).

The antibody–probe complexes were applied using 15 μL for each sample and left for 15 min for binding. Excess complexes were washed out with PBS and covered with another polyimide film. Samples were sandwiched by stainless steel frames and screw-clamped.

### 2.2. Sample Preparation for Dual-Labeling DXT

Sample preparation for dual-labeling DXT was performed using the method described previously [19] with minor modifications. The full-length human TRPV1 was inserted into pcDNA3.1 and a (His)_6_ sequence at the N-terminus and a FLAG sequence at the C-terminus were added. Expression of the TRPV1 protein was achieved in HEK293F cells, followed by cell surface biotinylation using sulfo-NHS-LC-biotin (Thermo Fisher, Waltham, MA, USA) as per the manufacturer’s guidelines. Subsequently, the TRPV1 protein underwent purification through a two-step process involving anti-FLAG affinity chromatography (Sigma-Aldrich, St. Louis, MO, USA) and Superdex 200 Increase size exclusion chromatography (Cytiva, Marlborough, MA, USA). The resulting peak fractions were concentrated to 0.1 mg/mL for downstream experiments.

To immobilize the biotinylated TRPV1 proteins onto the substrate, a 12.5 μm thick polyimide film (Du Pont-Toray, Tokyo, Japan) was first coated with cadmium and chromium (Cd/Cr) through evaporation. Subsequently, biotin-functionalized self-assembled monolayers (Biotin-SAM) were formed on the surface using Biotin-SAM Formation Reagent (Dojindo, Kumamoto, Japan). Streptavidin was then bound to the Biotin-SAM membrane. Twenty microliters of biotinylated TRPV1 (bearing N-terminal (His)_6_ and C-terminal FLAG tags, with extracellular biotinylation, at a concentration of approximately 0.1 mg/mL) were applied to the membrane and allowed to incubate at 4 °C for 6 h. Excess proteins were subsequently washed away with buffer. The gold–(His)_6_ antibody complexes and ZnO–FLAG antibody complexes were applied using 15 μL for each and left for 20 min for binding. Excess complexes were washed out with recording buffer (PBS (pH 7.4) with 5 mM n-decyl-β-D-maltoside (Sigma-Aldrich)). Ten microliters of recording buffer containing 10 μM capsaicin (Sigma-Aldrich), with or without 10 μM AMG9810 (Sigma-Aldrich), was applied. The sample chamber was covered with another layer of polyimide film, sandwiched by stainless steel frames and screw-clamped. The concentration of the antibody–probe complexes was optimized to obtain approximately 500–1000 trajectories per recording.

### 2.3. DXB and DXT Recording

DXB and DXT recording were performed using the method described previously [19]. For DXB measurement, time-resolved diffraction images were recorded at the Photon Factory Advanced Ring NW14A beamline. The diffraction data were recorded using a 2D photon-counting detector, PILATUS 100 K array (pixel size 172 × 172 µm^2^, max framing rate 300 Hz, readout time 2.3 ms, 20 bit counter depth; DECTRIS, Baden-Dättwil, Switzerland). The distance between the sample and the detector was set at 50 mm, and the beam size was adjusted to 250 μm × 250 μm. Exposure was performed with a 100 ms/frame rate, recording 2000 frames.

The temperature of the sample chamber was maintained at 25 °C utilizing the Peltier cooling-and-heating stage (Type 10084L, Japan Hightech, Fukuoka, Japan). An assessment of X-ray-induced damage involved comparing the decay constant distributions calculated from two groups: the first half and the second half of the data.

For DXT measurement, the gap of the insertion device U20 was set to 15 mm, and white X-rays with a peak energy of 17.7 keV and an energy bandwidth of 0.1 (photon flux of 10^13^ photons/s) were utilized [22]. The distance between the sample and the detector was adjusted to 95 mm, with a beam size of 100 μm × 250 μm. Data were captured at intervals of 12.5 ms per frame and measured for 5000 frames per spot. Recordings were conducted in triplicate for each sample, with a 0.2 mm distance between recordings.

### 2.4. Data Analysis for DXB

The intensity trajectories were extracted from each pixel around the Au (111) and ZnO (100), (002), (101) diffraction rings and analyzed. The data within the intermodular rectangular area of the detector were masked. Time course analysis of the diffracted photon signal, represented as *I*(*t*), yields insights into the movements of probe particles. To examine the fluctuations, the autocorrelation function (ACF) of photon intensity was computed for each pixel using the equation

ACF = <*I*(*t*)*I*(*t* + *τ*)>/<*I*(*t*)^2^>

where *I*(*t*) represents the number of diffracted photons as a function of time. *τ* is the delay time. To determine the decay constant (*T*), single-pixel ACFs are fitted to single exponential curves using the equation

ACF = *k* + *A* exp (−*Tt*),

where *k* is a constant and *A* is the amplitude. ACF curves that met the fitting criteria of *k* > 0, *A* > 0, and *T * > 0 were employed to elucidate the averaged ACF curves. These averaged curves were then fitted using a weighted least-squares method, with weight values determined by the standard error. Subsequently, the ACF decay constant distributions were subjected to statistical analysis utilizing the non-parametric Wilcoxon rank sum test. Statistical significance was denoted as *** *p* < 0.001.

### 2.5. Image Analysis for DXT

Image analysis for DXT was performed following the method described previously [19]. The obtained data were calibrated with the concurrently recorded intensity of incident X-ray (*I*_0_). Subsequently, each diffraction spot was tracked using TrackPy (v0.3.2) [23] after correcting the background noise. Trajectories were then analyzed using custom software developed within IGOR Pro version 9 (Wavemetrics, Lake Oswego, OR, USA). For the analysis, diffraction spots corresponding to Au (111), ZnO (100), (002), and (101) were utilized. The diffraction spots originating from fast-moving proteins exhibited a short time duration between appearance and disappearance in the observation area, whereas those from slow-moving proteins persisted for longer durations.

Protein dynamics were further analyzed utilizing a temporal mean squared displacement (MSD) algorithm to extract the local behavior of the protein over time. The MSD curves were fitted using the following function: *δ*^2^ (*t*) = *D_α_ t^α^* + 2*β*^2^. *D_α_* is the anomalous diffusion constant, a nonlinear relationship to time. *α* represents subdiffusion (1 > *α* > 0) or superdiffusion (*α* > 1), and *β* is a measurement error.

## 3. Results

**Figure 1:**
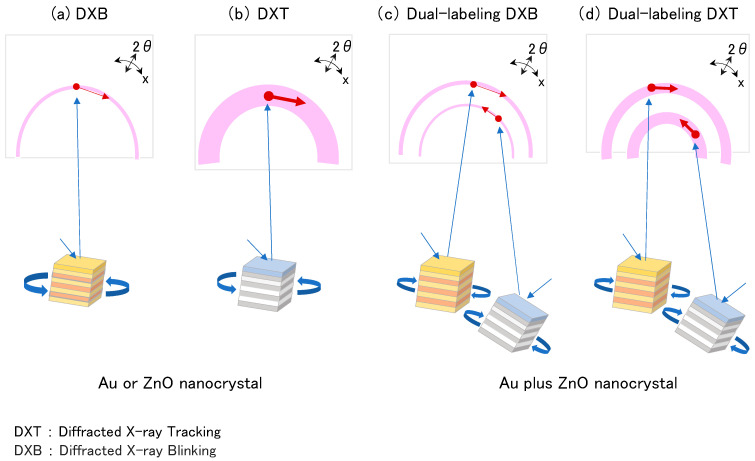
Diffracted X-ray blinking (DXB) and diffracted X-ray tracking (DXT). Schematic illustration of (**a**) DXB, (**b**) DXT, (**c**) dual-labeling DXB, and (**d**) dual-labeling DXT. The DXB and DXT utilize Au or ZnO nanocrystals for labeling, while dual-labeling DXB and dual-labeling DXT require two different nanocrystals which have different properties of the diffraction pattern. The blue arrows illustrate the generation of diffraction spots, and the red arrows show the motion of diffraction points.

The DXB and DXT techniques have been applied to understand the intramolecular dynamics of macromolecules, including ion channels, G protein-coupled receptors (GPCRs), and constitutive proteins [7,8,9]. In this study, we devised dual-labeling techniques for DXB and DXT, enabling simultaneous analysis of the dynamics at two independent domains of proteins under identical conditions (Figure 1).

Conventional DXB/DXT uses gold nanocrystals as molecular probes. Gold probes possess advantages in high X-ray diffraction sensitivity and the capability of forming covalent bonds with cysteine residues of the target protein. Gold particles are therefore extensively employed in various experiments. Due to the insufficient diffraction elicited by commercially available colloidal gold, we obtain in-house high-quality gold nanocrystals by the epitaxial growth of Au nanocrystals on KCl (100) substrates [24].

To achieve dual labeling in X-ray diffraction analysis, it is necessary to use second labeling probes with different diffraction characteristics from gold. We identified ZnO crystals as promising candidates for this role. ZnO crystals exhibit excellent diffraction patterns, high chemical stability, and favorable binding properties with proteins, rendering them a suitable labeling probe from a structural standpoint.

### 3.1. Motion Analysis of 5-HT_2A_ Serotonin Receptor on the Living Cells Using Dual-Labeling DXB Technique

**Figure 2:**
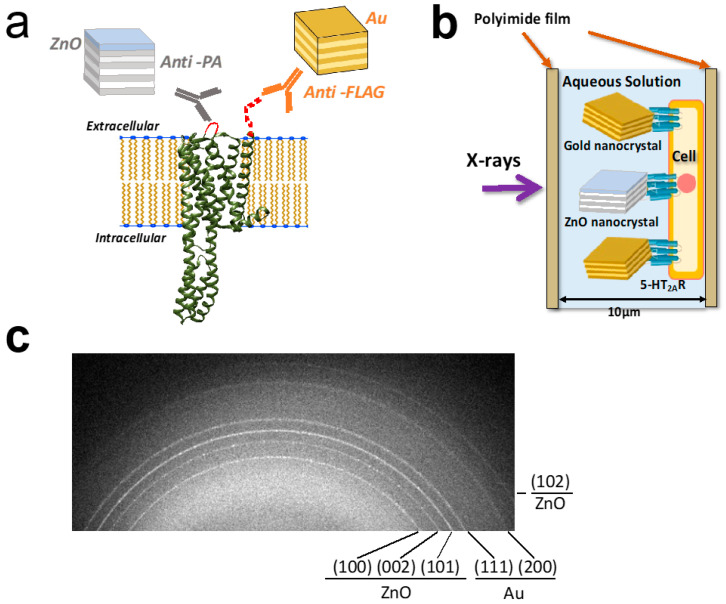
Dual-labeling DXB analysis of 5-HT_2A_ receptor in living cells. (**a**) Dual-labeling of the 5-HT_2A_R with gold and ZnO nanocrystals. The FLAG-tagged N-terminus and the PA-tagged second extracellular loop were specifically labeled by anti-FLAG antibody-decorated Au nanocrystals and anti-PA antibody-decorated ZnO nanocrystals, respectively. The 5-HT_2A_Rs were expressed on the HEK293 cells. (**b**) Dual-labeling living-cell DXB analysis. The HEK293 cells expressing 5-HT_2A_R were confluently cultured on the polyimide basement. After labeling with antibody–probe complexes, cells were covered with another polyimide film, and set into the sample holders. Time-resolved X-ray diffraction images were recorded. Considering the size differences between the proteins and the metal probes, multiple labeling on a single protein will not be possible. (**c**) Diffraction rings were obtained in this dual-labeling DXB analysis. Multiple diffraction rings were observed. The (100), (002), (101), and (102) rings originated from ZnO crystals, and (111) and (200) rings originated from Au crystals were distinguishable each other.

In this study, we applied a dual-labeling technique for the simultaneous monitoring of two distinct domains of the 5-HT_2A_R on living HEK293 cells. The FLAG tag at the N-terminus was labeled with Au-bound FLAG antibodies, while the PA tag at the second extracellular loop was labeled with ZnO-bound PA tag antibodies (Figure 2a). The PA tag antibodies, NZ-1, can recognize even loop structures and are well suited for the detection of loops of membrane proteins. Cells expressing 5-HT_2A_R were cultured on poly-L-lysine-coated polyimide films (Figure 2b). Diffraction rings from Au and ZnO were successfully obtained (Figure 2c). The diffraction rings from hexagonal ZnO crystal planes of (100), (002), (101), and (102) can be distinguished from those of the face-centered cubic Au crystal planes of (111) and (200). Their dynamics were statistically analyzed.

**Figure 3:**
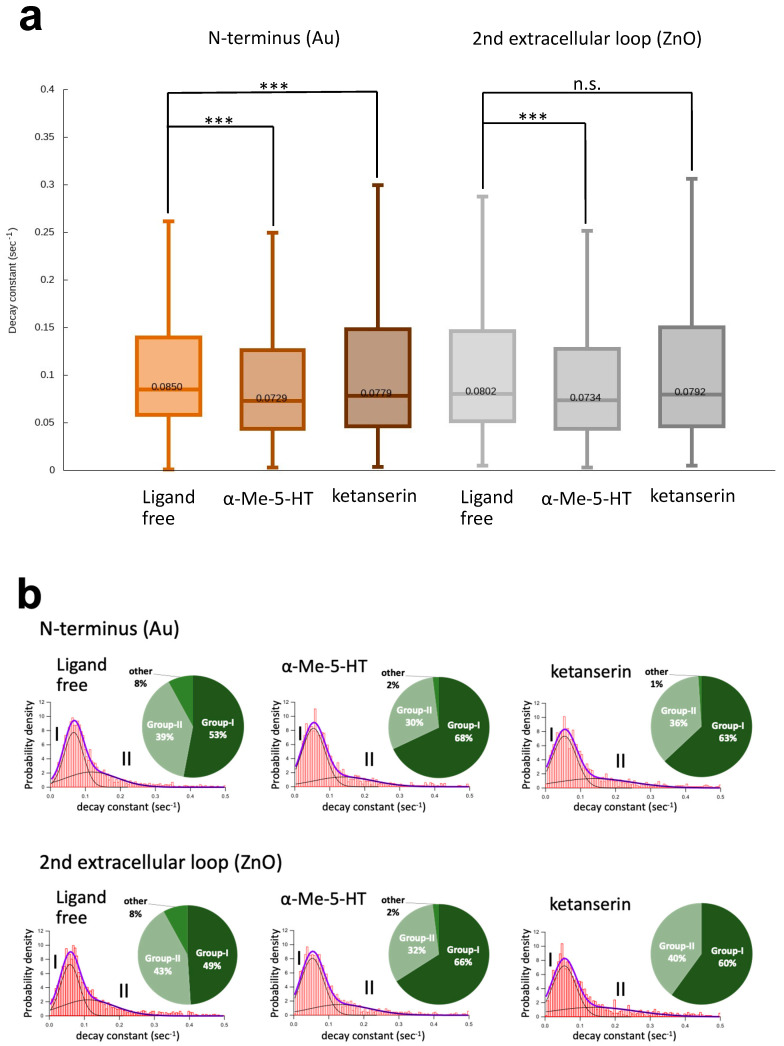
Dynamics of N-terminus and the second extracellular loop of 5-HT_2A_R on living-cells. (**a**) ACF decay constant calculated from ROI. The box plots represent the minimum, 25th percentile, 50th percentile, 75th percentile, and maximum. (Left) The motion of Au-labeled N-terminus was suppressed by both alpha methylserotonin (α-Me-5-HT) and ketanserin, and (right) the motion of ZnO-labeled second extracellular loop was suppressed by α-Me-5-HT but was not significant by ketanserin. The non-parametric Wilcoxon rank sum test was performed to compare two samples. *** *p* < 0.001. n.s., not significant. (**b**) Histograms of decay constants. Each histogram was fitted by two Gaussian curves (group-I and group-II). Inset circular graphs represent percentages of the fitted area. Thick purple lines in each histogram represent a sum of two Gaussian curves, illustrating the successful fitting of the experimental data.

Decay constants were calculated for each pixel from the region of interest (ROI) of the diffraction rings. Results are presented as box plots showing the minimum, 25th percentile, 50th percentile, 75th percentile, and maximum (Figure 3a). Under ligand-free conditions, the 50th percentile of the N-terminus was 0.0850/s, while that of the second extracellular loop was 0.0802/s, suggesting that the steady-state motion of the N-terminus was slightly higher than that of the second extracellular loop. This is probably because the N-terminal domain of the 5-HT_2A_R has a predominantly disordered 75-amino acid sequence. At the N-terminus, the motion was reduced by both an agonist alpha-methyl serotonin (α-Me-5-HT) and an antagonist ketanserin. The movement at the second extracellular loop was also reduced by α-Me-5-HT, but no significant suppression was observed with ketanserin.

The distribution of decay constants was illustrated by histograms fitted with two Gaussian curves: the low-mobility group (group-I) in the left curves and the high-mobility group (group-II) in the right curves (Figure 3b). Under ligand-free conditions, the populations of group-I and group-II are equivalent, or there is a slight tendency toward group-I in both positions. Ligand application drastically changed the population ratio. The α-Me-5-HT increased the population of group-I to 68% at the N-terminus and to 66% at the second extracellular loop (Figure 3b, middle columns). The ketanserin also increased the group-I population to 63% at the N-terminus and 60% at the second loop (Figure 3b, right columns). The percentage of motion contraction to group-1 is higher in α-Me-5-HT than in ketanserin in both positions. These results may support the hypothesis that the GPCRs in the native state exist in an equilibrium between active and inactive states and that the active ligands converge them into one structure and suppress structural fluctuations [25,26,27].

### 3.2. Intracellular Domain Dynamics of TRPV1 Channel Visualized by the Dual-Labeling DXT Technique

**Figure 4:**
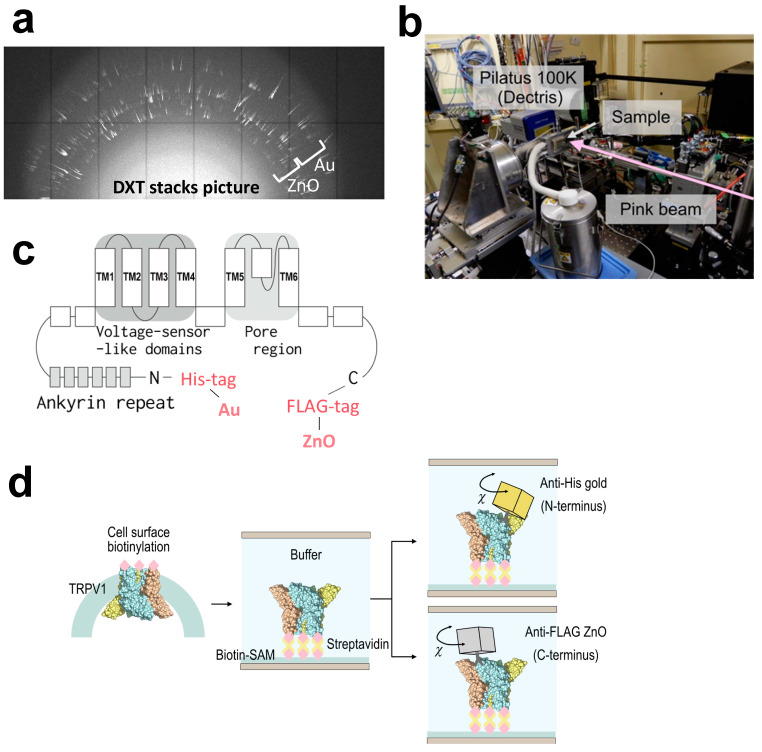
Dual-labeling DXT analysis of TRPV1 cytoplasmic domains. (**a**) Trajectories of diffraction spots generated from Au and ZnO nanocrystals. (**b**) Experimental setup for the DXT measurement at the Photon factory AR-NW14A beamline. (**c**) Two-dimensional structure of TRPV1 and inserted tags. (**d**) Sample preparation for dual-labeling DXT. N-terminally (His)_6_-tagged, C-terminally FLAG-tagged, and biotinylated TRPV1 was immobilized through a biotin-SAM (self-assembled monolayer) formed on the polyimide films. Gold-conjugated (His)_6_-antibodies and ZnO-conjugated FLAG antibodies were used to label the N- and C-terminus of TRPV1, respectively. The chamber was sandwiched by stainless steel frames and screw-clamped. Considering the size differences between the proteins and the metal probes, multiple labeling on a single protein will not be possible.

We conducted a simultaneous measurement of the N- and C-terminal motions of the purified TRPV1 protein using the DXT technique (Figure 4a,b). TRPV1 functions as a homotetramer channel, composed of four subunits, each featuring six transmembrane segments and a pore-forming region. The pore is created by the assembly of transmembrane segment 5 (S5), a pore loop, and transmembrane segment 6 (S6), enclosed by a voltage sensor-like domain comprising a bundle of four transmembrane helices (S1–S4). Notably, both the N- and C-termini are situated intracellularly, and an ankyrin repeat domain is present at the N-terminus (Figure 4c). The most controversial issue regarding the cryo-EM structures is the presentation of the capsaicin-bound structure as a “closed form”, with only the lower gate opening among the dual gate system [28]. Subsequent cryo-EM studies demonstrated the “open form” by applying capsaicin and heat simultaneously [29], but raised a puzzling question of why capsaicin alone fails to induce the open conformation. Our previous studies using the DXT technique suggested that the twisting motion at the extracellular domains may facilitate gate opening by capsaicin [18,19].

The N-terminally (His)_6_-tagged and C-terminally FLAG-tagged TRPV1 proteins were transiently expressed in HEK293F cells in suspension culture, and their extracellular domain was biotinylated using the surface biotinylation technique [30]. Purified TRPV1 protein was bound by a biotin–streptavidin reaction to Cd/Cr-coated polyimide films, on which biotin-SAM (self-assembled monolayers) has been generated in advance. The motions of the N- and C-termini were monitored using the gold-(His)_6_-antibody conjugates and ZnO-FLAG antibody conjugates, respectively (Figure 4d). After many trials with various ZnO resources, we selected “zinc oxide nanoparticles” from EM-Japan with an average diameter of 500 nm due to their superior performance pertaining to diffraction pattern, size, and stability. In order to obtain the trajectories of diffraction spots, the beam source of KEK NW-14A was adjusted to 17.7 keV peak energy exhibiting a wide energy bandwidth. Data were recorded using a Pilatus 100 K camera at 12.5 ms per frame (Figure 4b). We successfully obtained diffraction data both from Au and ZnO nanocrystals simultaneously. The stacking data showed that they were well separated and could be analyzed independently (Figure 4a). An innermost white halo was produced by the buffer components in the recording area, but this did not affect the data analysis.

**Figure 5:**
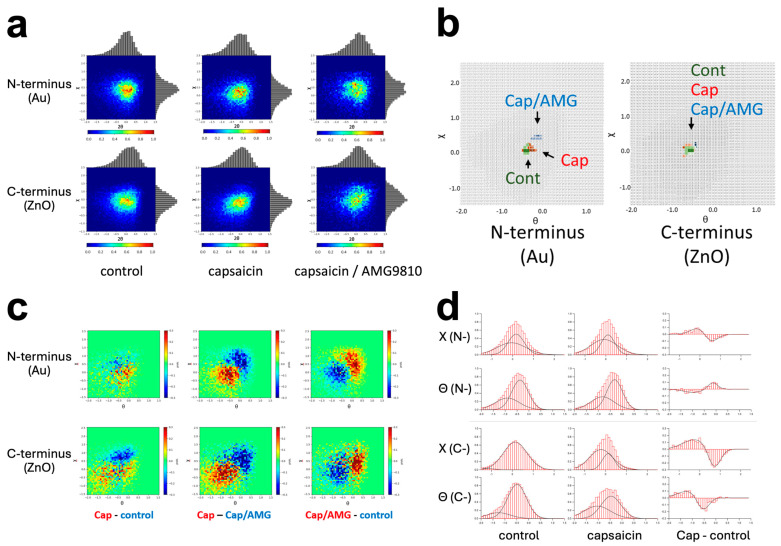
Capsaicin-induced dynamics of N- and C-terminus of TRPV1 obtained by the dual-labeling DXT. (**a**) Two-dimensional motion histograms with θ-χ coordinates, along with their projected 1D histograms. (**b**) Superposed maps representing hotspots in motion for N-terminus (left) and C-terminus (right). (**c**) Subtraction heat maps of capsaicin minus control (left), capsaicin minus Cap/AMG (middle), and Cap/AMG minus control (right), for the N-terminus (upper row) and the C-terminus (lower row). (**d**) Distribution maps of each motion component (χ and θ) of control and capsaicin, and their subtraction maps.

The trajectory data were divided into two axes, twisting (χ) and bending (θ), and they were analyzed separately. Motion was represented by two-dimensional (2D) histograms of θ-χ coordinate, together with their projected one-dimensional (1D) histograms (Figure 5a). In the control condition, the population in the 2D distribution of the C-terminus seems slightly more dispersed than that of the N-terminus (Figure 5a, left columns). Indeed, the full width at half maximum (FWHM) values of the 1D histograms were slightly larger for the C-terminus than the N-terminus (FWHM of χ: 1.154 (N) and 1.365 (C); FWHM of θ: 1.008 (N) and 1.029 (C)), indicating that the motion of the C-terminus involves different motion components (Supplementary Table S1).

Capsaicin increased the θ value of the N-terminus. The peak position in the 1D histograms was shifted from 0.105 (control) to 0.040 (capsaicin) in the χ coordinate, and from −0.491 (control) to −0.440 (capsaicin) in the θ coordinate (Figure 5a, upper left and middle; Supplementary Tables S1 and S2). This accompanies the movement of hotspots, as shown in the superposition maps (Figure 5b, left). This was in good contrast to the capsaicin-induced motion change at the C-terminus, in which the coordinates of the hotspots did not show significant change among the three experimental conditions (Figure 5b, right). Consequently, the 2D histograms of the C-terminus with capsaicin showed an oval shape along the diagonal line (Figure 5a, lower middle), suggesting the appearance of slower-moving populations. The capsaicin-induced motion suppression was recovered at both the N- and C-terminus by AMG9810, as demonstrated in the superposed hotspot maps (Figure 5b).

Subtraction maps visualized the motion shift between two experimental conditions. The “capsaicin minus control” (Figure 5c, left columns) showed a moderate level of motion suppression, as indicated by the population shift toward the lower left corner. This motion retardation was recovered by AMG9810, as shown by the “Cap/AMG minus control” distributions (Figure 5c, right columns), in which these two populations were mixed. The motion shift was more clearly observed at the N-terminus than at the C-terminus, which may reflect the difference in hotspot shift observed in Figure 5b. The AMG9810 triggered enhanced motion in the C-terminus (Figure 5c, lower right), consistent with our previous results [19]. We speculate that AMG9810 inhibits the endogenous rotational movement in TRPV1 and extracts only the CW directional movement [18]. We analyzed distribution histograms of each motion component (χ and θ) for the both control and capsaicin. The subtraction maps revealed a general suppression of most components by capsaicin, except for an enhancement in the motion component of θ at the N-terminus (Figure 5d).

The dual-labeling DXT technique successfully elucidated the ligand-induced N- and C-terminal motions of TRPV1 independently. Recently, we performed motion measurements of TRPV1 domains at a fast frame rate of 100 μs/frame, revealing capsaicin-induced rapid fluctuations at the C-terminus [19]. Expanding the application of dual-labeling DXT to various conditions will be helpful for further validation.

## 4. Discussion

In contrast to the wide choice of structure determination methods, techniques for the real-time observation of intramolecular protein dynamics remain extremely limited. To address this problem, we have used the DXT method, which has the highest spatiotemporal resolution among all existing single-molecule measurement techniques. DXT has been applied to analyze the intramolecular dynamics of various membrane proteins [8].

As X-rays easily penetrate protein molecules, labeling probes should interact strongly with X-rays. Gold has been used as an excellent probe due to its high sensitivity to X-rays and its ability to form covalent bonds with cysteine residues on the protein surface. In order to obtain sufficient diffraction from the gold nanocrystals, we have developed a method to obtain high-quality gold nanocrystals that are epitaxially grown on the KCl crystal [24].

The real-time measurement of multiple domain movements is the most promising approach for understanding the functional mechanisms of target proteins. In this study, we found that zinc oxide crystals are suitable labeling probes due to their high-quality diffraction spots, high chemical stability, and favorable protein binding. The general lattice constants are approximately a-axis 3.25 Å and c-axis 5.2 Å for zinc oxide, and a-axis 4.08 Å for gold [31]. The diffraction rings of ZnO (100), (002), (101), (102), and Au (111) and (200) were distinguishable, allowing for an independent analysis of each domain’s motion. We used the diffraction information of ZnO (100), (002), (101), and Au (111) for data analysis.

The influence of antibody flexibility should be carefully considered [32]. The molecular surface cysteines are predominantly situated near the Fab-Fc junctions in IgG molecules. Therefore, the significance of IgG flexibility in DXT measurement, when coupled with the statistical analysis of thousands of diffraction data, may not be so significant. However, to avoid the influence of IgG flexibility, our future studies will involve the use of Fab fragments or much smaller molecules, such as VHH (variable domain of the heavy chain of a heavy-chain antibody), for conjugation to probes [33,34].

To obtain sufficient diffraction, the diameters of the nanocrystals used in this study were 40–80 nm for gold and an average of 500 nm for zinc oxide. Therefore, the risk of steric hindrance should be considered when all epitopes are labeled by probes. To address these concerns, we reduced the labeling density to obtain adequate diffraction counts per measurement. Since it is estimated that less than 10% of the existing epitopes are labeled, significant steric hindrance is effectively avoided. Considering the size differences between the proteins and the metal probes, multiple labeling on a single protein would not be possible. The detector performance and signal strength define the detection limit of DXT. Our previous experiments demonstrated a subtle size-dependent suppression of protein motion [35]. Experiments involving the use of reduced sizes of Au and ZnO nanocrystals, within the improved DXT technique, are anticipated.

The protein dynamics, particularly those involving domain movements, are typically recognized in a nanosecond to millisecond order [36]. By selecting an adequate recording speed during data acquisition and applying the lifetime filtering method, which involves grouping and analyzing data based on the duration of diffraction spots, we successfully separated and extracted motion components with different velocities [18,37]. Dual-labeling DXB/DXT allows a motion comparison of remote domains of a protein under identical experimental conditions. This approach is further bolstered by integrating time-lapse experiments, such as stopped flow ligand application systems, or by observing the response to physical stimuli such as light, heat, and tension. This comprehensive methodology will provide a robust understanding of the protein dynamics and contribute to an accurate interpretation of the experimental results.

## Data Availability

The data that support the findings of this study are available in the article and the Supplementary Materials. Additional data related to this article are available on request from the corresponding author.

## Supplementary Materials

The following supporting information can be downloaded at: https://www.mdpi.com/article/10.3390/membranes14040075/s1, Supplementary Table S1: Fitting parameters for the 1D histograms (N-terminus); Supplementary Table S2: Fitting parameters for the 1D histograms (C-terminus).
