# Supplementary material for: Simultaneous Recording of Remote Domain Dynamics in Membrane Proteins Using the Double-Labeled DXB/DXT Technique
Source: Membranes (Basel). 2024 Mar 27;14(4):75. doi: 10.3390/membranes14040075 (PMC11052370; doi:10.3390/membranes14040075)
Supplement: Supplementary file 1 [file membranes-14-00075-s001.zip › membranes-2834729-supplementary.pdf]

Supplementary tables

Supplementary Table 1. Fitting parameters for the 1D histograms (N-terminus)

	Control			Capsaicin			AMG9810 (+Cap)		
χ coordinate									
Location *	0.105	+/-	0.008	0.040	+/-	0.009	0.294	+/-	0.01
FWHM *	1.154	+/-	0.025	1.065	+/-	0.027	1.309	+/-	0.03
Area	0.921	+/-	0.023	0.901	+/-	0.026	0.963	+/-	0.03
θ coordinate									
Location *	-0.491	+/-	0.011	-0.440	+/-	0.011	-0.256	+/-	0.01
FWHM *	1.008	+/-	0.032	0.974	+/-	0.033	1.038	+/-	0.04
Area	0.918	+/-	0.033	0.912	+/-	0.035	0.917	+/-	0.04

*: Log(abs(displacement) [mrad])

Supplementary Table 2. Fitting parameters for the 1D histograms (C-terminus)

	Control			Capsaicin			AMG9810 (+Cap)		
χ coordinate									
Location *	0.127	+/-	0.008	-0.051	+/-	0.013	0.306	+/-	0.01
FWHM *	1.365	+/-	0.026	1.120	+/-	0.038	1.161	+/-	0.03
Area	0.966	+/-	0.023	0.918	+/-	0.036	0.895	+/-	0.03
θ coordinate									
Location *	-0.553	+/-	0.009	-0.641	+/-	0.012	-0.262	+/-	0.02
FWHM *	1.029	+/-	0.027	1.267	+/-	0.039	1.247	+/-	0.07
Area	0.918	+/-	0.028	0.980	+/-	0.038	0.983	+/-	0.07

*: Log(abs(displacement) [mrad])
